# Efficacy of canakinumab in patients with Still’s disease across different lines of biologic therapy: real-life data from the International AIDA Network Registry for Still’s Disease

**DOI:** 10.3389/fmed.2023.1256243

**Published:** 2023-12-12

**Authors:** Antonio Vitale, Valeria Caggiano, Petros P. Sfikakis, Lorenzo Dagna, Giuseppe Lopalco, Gaafar Ragab, Francesco La Torre, Ibrahim A. Almaghlouth, Maria Cristina Maggio, Jurgen Sota, Abdurrahman Tufan, Andrea Hinojosa-Azaola, Florenzo Iannone, Roberta Loconte, Katerina Laskari, Haner Direskeneli, Piero Ruscitti, Maria Morrone, Henrique A. Mayrink Giardini, Alexandros Panagiotopoulos, Ilenia Di Cola, Eduardo Martín-Nares, Sara Monti, Ludovico De Stefano, Rıza Can Kardas, Rahime Duran, Corrado Campochiaro, Alessandro Tomelleri, Abdulaziz Mohammed Alabdulkareem, Carla Gaggiano, Maria Tarsia, Elena Bartoloni, Mery Romeo, Mohamed A. Hussein, Ahmed Hatem Laymouna, Isabele Parente de Brito Antonelli, Marilia Ambiel Dagostin, Lampros Fotis, Sara Bindoli, Luca Navarini, Fatma Alibaz-Oner, Gizem Sevik, Micol Frassi, Francesco Ciccia, Daniela Iacono, Francesca Crisafulli, Piero Portincasa, Nour Jaber, Perla Ayumi Kawakami-Campos, Ewa Wiesik-Szewczyk, Annamaria Iagnocco, Gabriele Simonini, Paolo Sfriso, Alberto Balistreri, Roberto Giacomelli, Giovanni Conti, Bruno Frediani, Claudia Fabiani, Luca Cantarini

**Affiliations:** ^1^Department of Medical Sciences, Surgery and Neurosciences, Research Center of Systemic Autoinflammatory Diseases and Behçet's Disease Clinic, University of Siena, Siena, Italy; ^2^Azienda Ospedaliero-Universitaria Senese, European Reference Network (ERN) for Rare Immunodeficiency, Autoinflammatory and Autoimmune Diseases (RITA) Center, Siena, Italy; ^3^Joint Academic Rheumatology Program, Medical School, National and Kapodistrian University of Athens, Athens, Greece; ^4^Division of Immunology, Transplants and Infectious Diseases, Università Vita-Salute San Raffaele, Milan, Italy; ^5^Unit of Immunology, Rheumatology, Allergy and Rare Diseases, IRCCS Ospedale San Raffaele, Milan, Italy; ^6^Department of Precision and Regenerative Medicine and Ionian Area (DiMePRe-J) Policlinic Hospital, University of Bari, Bari, Italy; ^7^Rheumatology and Clinical Immunology Unit, Internal Medicine Department, Faculty of Medicine, Cairo University, Giza, Egypt; ^8^Faculty of Medicine, Newgiza University, 6th of October City, Egypt; ^9^Department of Pediatrics, Pediatric Rheumatology Center, Giovanni XXIII Pediatric Hospital, University of Bari, Bari, Italy; ^10^Rheumatology Unit, Department of Medicine, College of Medicine, King Saud University, Riyadh, Saudi Arabia; ^11^College of Medicine Research Center, College of Medicine, King Saud University, Riyadh, Saudi Arabia; ^12^University Department of Health Promotion, Mother and Child Care, Internal Medicine and Medical Specialties (PROMISE) "G. D'Alessandro", University of Palermo, Palermo, Italy; ^13^Department of Internal Medicine, Division of Rheumatology, Gazi University Hospital, Ankara, Türkiye; ^14^Department of Immunology and Rheumatology, Instituto Nacional de Ciencias Médicas y Nutrición Salvador Zubirán, Mexico City, Mexico; ^15^Joint Academic Rheumatology Program, The First Department of Propaedeutic and Internal Medicine, School of Medicine, National and Kapodistrian University of Athens, Athens, Greece; ^16^Department of Internal Medicine, Division of Rheumatology, Marmara University, Faculty of Medicine, Istanbul, Türkiye; ^17^Rheumatology Unit, Department of Biotechnological and Applied Clinical Sciences, University of L'Aquila, L'Aquila, Italy; ^18^Rheumatology Division, Faculdade de Medicina, Hospital das Clínicas, Universidade de São Paulo, São Paulo, Brazil; ^19^Division of Rheumatology, Fondazione IRCCS Policlinico San Matteo, Pavia, Italy; ^20^Department of Internal Medicine and Therapeutics, Università di Pavia, Pavia, Italy; ^21^, Department of Molecular Medicine and DevelopmentClinical Pediatrics, University of Siena, Siena, Italy; ^22^Rheumatology Unit, Department of Medicine and Surgery, University of Perugia, Perugia, Italy; ^23^Pediatric Nephrology and Rheumatology Unit, Azienda Ospedaliera Universitaria (AOU), "G. Martino", Messina, Italy; ^24^Department of Pediatrics, Attikon General Hospital, National and Kapodistrian University of Athens, Zografou, Greece; ^25^Rheumatology Unit, Department of Medicine, University of Padua, Padua, Italy; ^26^Clinical and Research Section of Rheumatology and Clinical Immunology, Fondazione Policlinico Campus Bio-Medico, Rome, Italy; ^27^Rheumatology, Immunology and Clinical Medicine Unit, Department of Medicine, School of Medicine, University of Rome “Campus Biomedico”, Rome, Italy; ^28^Spedali Civili and Department of Clinical and Experimental Sciences, Rheumatology and Clinical Immunology, University of Brescia, European Reference Network (ERN) for Rare Immunodeficiency, Autoinflammatory and Autoimmune Diseases (RITA) Center, Brescia, Italy; ^29^Department of Precision Medicine, University of Campania "Luigi Vanvitelli", Naples, Italy; ^30^Clinica Medica "A. Murri", Division of Internal Medicine, Department of Precision and Regenerative Medicine and Ionian Area (DiMePre-J), University of Bari Aldo Moro, Bari, Italy; ^31^Department of Ophthalmology, Instituto Nacional de Ciencias Médicas y Nutrición Salvador Zubirán, Mexico City, Mexico; ^32^Department of Internal Medicine, Pneumonology, Allergology and Clinical Immunology, Central Clinical Hospital of the Ministry of National Defense, Military Institute of Medicine, National Research Institute, Warsaw, Poland; ^33^Academic Rheumatology Centre, Dipartimento Scienze Cliniche e Biologiche, Università degli Studi di Torino, Torino, Italy; ^34^NEUROFARBA Department, Rheumatology Unit, Meyer Children's Hospital IRCCS, University of Florence, Florence, Italy; ^35^Bioengineering and Biomedical Data Science Lab, Department of Medical Biotechnologies, University of Siena, Siena, Italy; ^36^Ophthalmology Unit, Department of Medicine, Surgery and Neurosciences, University of Siena, Siena, Italy

**Keywords:** AOSD, AutoInflammatory diseases, rare diseases, personalized medicine, treatment

## Abstract

**Introduction:**

The effectiveness of canakinumab may change according to the different times it is used after Still’s disease onset. This study aimed to investigate whether canakinumab (CAN) shows differences in short- and long-term therapeutic outcomes, according to its use as different lines of biologic treatment.

**Methods:**

Patients included in this study were retrospectively enrolled from the AutoInflammatory Disease Alliance (AIDA) International Registry dedicated to Still’s disease. Seventy-seven (51 females and 26 males) patients with Still’s disease were included in the present study. In total, 39 (50.6%) patients underwent CAN as a first-line biologic agent, and the remaining 38 (49.4%) patients were treated with CAN as a second-line biologic agent or subsequent biologic agent.

**Results:**

No statistically significant differences were found between patients treated with CAN as a first-line biologic agent and those previously treated with other biologic agents in terms of the frequency of complete response (*p* =0.62), partial response (*p* =0.61), treatment failure (*p* >0.99), and frequency of patients discontinuing CAN due to lack or loss of efficacy (*p* =0.2). Of all the patients, 18 (23.4%) patients experienced disease relapse during canakinumab treatment, 9 patients were treated with canakinumab as a first-line biologic agent, and nine patients were treated with a second-line or subsequent biologic agent. No differences were found in the frequency of glucocorticoid use (*p* =0.34), daily glucocorticoid dosage (*p* =0.47), or concomitant methotrexate dosage (*p* =0.43) at the last assessment during CAN treatment.

**Conclusion:**

Canakinumab has proved to be effective in patients with Still’s disease, regardless of its line of biologic treatment.

## 1 Introduction

The treatment of Still’s disease has advanced remarkably in the last few years, with interleukin 1(IL-1) inhibition representing an effective and safe treatment option in patients with persistent inflammation after adequate glucocorticoid (GC) treatment (1). The association of conventional disease-modifying antirheumatic drugs (cDMARDs), especially methotrexate, may be useful when some inflammatory manifestations persist despite systemic improvement, such as in patients with polyarticular involvement and those with persistent arthritis or arthralgia (2–4). While the IL-6 antagonist tocilizumab has been proven to be effective in treating severe and persistent Still’s disease (5, 6), tumor necrosis factor inhibitors have been proven to have some role but seem to be replaced by anti-IL-1 and anti-IL-6 agents in terms of systemic efficacy (6).

Inhibition of IL-1 accounts for the most commonly employed biologic treatment for Still’s disease at present; however, many therapeutic aspects need to be elucidated, including the best timing for the start of treatment. In this regard, a window of opportunity has been proposed for patients with systemic juvenile idiopathic arthritis (sJIA), the pediatric counterpart of Still’s disease, to be treated with the IL-1 receptor antagonist anakinra. In particular, a disease duration of ≤3.9 years was suggested as a clinical condition associated with complete response (7). Later, no window of opportunity was identified in patients treated with anakinra for adult-onset Still’s disease (AOSD) (8). Therefore, this study aimed to investigate whether the monoclonal IL-1β inhibitor canakinumab (CAN) shows differences in short- and long-term therapeutic outcomes according to the timing of its use in patients with Still’s disease in terms of different lines of biologic treatment.

## 2 Materials and methods

Patients included in this study were drawn from the AutoInflammatory Disease Alliance (AIDA) international registry dedicated to Still’s disease (9). Data were collected retrospectively.

Patients with Still’s disease were classified according to internationally accepted criteria [Yamaguchi and/or Fautrel for adult patients (10, 11); the International League of Associations for Rheumatology (ILAR) and/or Pediatric Rheumatology International Trials Organization (PRINTO) criteria for patients aged <16 years (12, 13)]. The enrollment period was between June 2021 and March 2023; the *Index Date* to enter the study corresponded to the date at the time of enrollment in the AIDA registry; the observational period ranged from the time at disease onset to the last follow-up assessment. We meshed both patients with pediatric and adult disease onset, as these conditions share several clinical and biological features and are increasingly considered the same entity arising at different ages (14).

Inclusion criteria were as follows: enrollment in the AIDA international registry dedicated to Still’s disease (9); treatment with CAN during the patient’s history; fulfillment of at least one set of criteria for adult patients (10, 11) or for pediatric patients (12, 13); patients’ consent and/or assent for the use of their data. Exclusion criteria: lack of data about the line of biologic treatment.

This study aimed to examine the differences in CAN effectiveness according to its use as different lines of biologic treatment. The endpoints of the study were a statistically significant difference between patients treated with CAN as a first-line biologic agent and those previously treated with other biologic agents in terms of the frequency of complete response, partial response, failure, glucocorticoid-sparing effect, need for concomitant cDMARDs, occurrence of relapses during treatment, and frequency of discontinuation due to a lack or loss of efficacy.

The patients were stratified according to the line of biologic treatment involving CAN, and those treated with CAN as a first-line biologic agent were compared with patients previously administered at least one other biologic agent.

*Complete response* was defined as the resolution of all the manifestations and laboratory inflammatory features that patients presented with at baseline. *Partial response* consisted of the persistence or recurrence of clinical manifestations with a remarkable decrease in their severity/frequency, with inflammatory laboratory parameters normalized or only slightly increased. A *failure group* included patients with no reduction in the frequency or severity of Still’s disease manifestations, despite therapeutic adjustments (CAN dosage increase or cDMARDs association). Complete response, partial response, and failure referred to the global efficacy of CAN observed during the first 12 months after the start of CAN. A *relapse* was defined as the reappearance of Still’s disease-related clinical manifestations during CAN. *Lack of efficacy* concerned patients who experienced failure as early as the first 3–6 months of CAN treatment, and *loss of efficacy* concerned patients who experienced immediate clinical benefit after the introduction of CAN, with subsequent failure occurring after a clinical benefit of at least six months. Definitions have been retraced from other examples in the literature (3, 15, 16).

The glucocorticoid-sparing effect was tested by evaluating the number of patients who discontinued GCs and daily prednisone (or equivalent) dosage at 3-month visit and at the last assessment during CAN treatment.

This study was approved by the Ethics Committee of Azienda Ospedaliero Universitaria Senese, Siena, Italy (AIDA Project; Ref. N. 14,951), as part of the AIDA program (9). The study protocol conformed to the tenets of the Declaration of Helsinki, and informed consent was obtained from all patients or their next of kin at the time of recruitment to the AIDA Registry dedicated to Still’s disease.

### 2.1 Statistical analysis

Descriptive statistics included mean, standard deviation, median, and interquartile range (IQR) values, according to the data distribution tested using the Shapiro–Wilk test. For qualitative data, comparisons were performed using the Chi-square test or Fisher’s exact test, depending on the number of samples. For quantitative data, the Student’s *t*-test or Mann–Whitney U test was used for pairwise comparisons, as required. Multinomial logistic regression was used to identify any association between clinical outcomes (complete response/partial response/failure) as the dependent variable and disease duration at the start of CAN as an independent variable. The significance level was set at 95% (*p* < 0.05), and all the tests were two-sided. Statistical analyses were conducted using STATA 17/MP2 software (StataCorp. 2021. Stata Statistical Software: Release 17. College Station, TX, StataCorp LLC).

## 3 Results

A total of 77 patients (51 female and 26 male) were included in the present study. Of them, 43 (55.8%) presented with a systemic pattern of Still’s disease, 18 (23.4%) showed a chronic articular disease course, and 16 (20.8%) were not classified according to the disease course due to the short follow-up period. The ethnicities of the patients were as follows: 64 (83.1%) Caucasian, 3 (3.9%) Hispanic, 2 (2.6%) Arab, and 1 (1.3%) Asian. Ethnicity was not reported in seven cases.

The mean age at disease onset was 28.0 ± 16.6 years; 22 (28.6%) patients experienced disease onset prior to the age of 16 years old. The mean age at enrollment was 33.4 ± 17.6 years. The median disease duration at the start of CAN was 9 (33) months among patients being treated with their first biologic agent and 22 (52) months among patients previously administered other biologic treatments. Table 1 describes the demographic and clinical features of the patients, according to the line of biologic treatment of CAN.

**Table 1:** Clinical and laboratory features of the 77 patients enrolled in this study, divided into two groups according to the line of biologic treatment with canakinumab.

	First-line (39 patients)	Second-line or subsequent biologic agent(38 patients)	*p*-value
Sex (female/male)	26/13	25/13	>0.99
Age at disease onset, mean ± SD	27.1 ± 16.9	28.9 ± 16.5	0.67
Age at diagnosis, mean ± SD	27.7 ± 17.1	30.8 ± 16.2	0.44
Age at disease onset <16 years, *n* (%)	13 (33.3)	9 (23.7)	0.49
Age at start of CAN <16 years, *n* (%)	10 (25.6)	6 (15.8)	0.43
Disease duration at start of CAN, median (IQR), months	9 (33)	22 (52)	0.01
Number of relapse/year during CAN, median (IQR)	3 (2)	2 (2)	0.33
Systemic/Chronic articular disease course	19/7	24/11	0.92
Not classified disease course	8 (20.5)	4 (10.5)	0.37
Clinical features observed during relapses preceding CAN, *n* (%)			
Pharyngitis	21 (53.8)	18 (47.4)	0.73
Salmon-colored rash	30 (77)	23 (60.5)	0.19
Atypical rash	9 (23.1)	7 (18.4)	0.82
Splenomegaly	9 (23.1)	15 (39.5)	0.19
Liver involvement	13 (33.3)	9 (23.7)	0.49
Arthralgia	36 (92.3)	29 (76.3)	0.1
Arthritis	20 (52.3)	18 (47.4)	0.91
Lymphadenopathy	16 (41)	20 (52.6)	0.43
Pneumonia	2 (5.1)	1 (1.3)	>0.99
Pleuritis	7 (17.9)	5 (13.2)	0.79
Pericarditis	8 (20.5)	5 (13.2)	0.58
Peritonitis	1 (2.6)	0 (0)	>0.99
Abdominal pain	6 (15.4)	6 (15.8)	>0.99
Clinical classification criteria, *n* (%)			
Yamaguchi et al. criteria	22/29 (75.9)	20/32 (62.5)	0.4
Fautrel et al. criteria	18/29 (62.1)	13/32 (40.6)	0.16
ILAR criteria	8/10 (80)	4/6 (66.7)	>0.99
PRINTO criteria	10/10 (100)	6/6 (100)	>0.99
Laboratory features during the attack preceding CAN introduction			
ESR, mean ± SD	85.04 ± 34.1	71.17 ± 24.02	0.09
CRP, median (IQR)	15 (15.76)	13 (30.8)	0.74
Ferritin serum level, median (IQR)	1381.5 (2755)	952 (4975.2)	0.51
Leukocytosis	24 (61.5)	23 (60.5)	>0.99
WBC, median (IQR)	17,000 (3460)	16,190 (5640)	0.36
Neutrophils (%), mean ± SD	83.6 ± 9.2	80.4 ± 9.2	0.23
Abnormal liver function tests [*n* (%)]	13 (33.3)	13 (34.2)	>0.99

CAN, canakinumab; CRP, C reactive protein; ESR, erythrocyte sedimentation rate; ILAR, International League of Associations for Rheumatology; IQR, interquartile range; PRINTO, Pediatric Rheumatology International Trials Organization; SD, standard deviation; WBC, white blood cells.

A total of 39 (50.6%) patients were treated with CAN as a first-line biologic agent. The other 38 (49.4%) patients were treated with CAN as a second-line (*n* = 31, 40.3%), third-line (*n* = 3, 3.9%), or fourth-line (*n* = 4, 5.2%) biologic agent. Among patients previously treated with other biologic agents, the preceding biologic treatments were anakinra in 32 (84.2%) patients, tocilizumab in 11 patients (28.9%), infliximab in 3 patients (7.9%), and etanercept in 2 patients (5.3%).

The reasons leading to the discontinuation of previous biologic agents were reported in 30/53 cases as follows: no efficacy in 12 cases, adverse events in 6 cases, loss of efficacy in 4 cases, only partial response in 4 cases, bureaucratic reasons (off-label use) in 3 cases, and long-term remission followed by disease exacerbation in 1 case. Table 2 describes the treatment approaches selected before the initiation of CAN.

**Table 2:** Conventional disease-modifying anti-rheumatic drugs (cDMARDs) used prior to canakinumab introduction.

	First-line (39 patients)	Second-line or subsequent biologic agent (38 patients)	*p*-value
cDMARDs, *n* (%)			
Methotrexate	21 (53.8)	22 (57.9)	0.9
Cyclosporine A	3 (7.7)	2 (5.3)	>0.99
Hydroxycloroquine	3 (7.7)	2 (5.3)	>0.99
Leflunomide	1 (2.6)	1 (2.6)	>0.99

Patients are divided between those treated with canakinumab as a first-line biologic agent and those previously treated with other biologic agents.

The mean age of patients at the start of the CAN therapy was 30.7 ± 17.1 years. Patients were administered 4 mg/Kg every 4 weeks, with a maximum of 300 mg/every 4 weeks when the dosage/Kg was higher. The median treatment duration was 19 months (IQR, 20 months) (range, 0–83 months).

**Figure 1:**
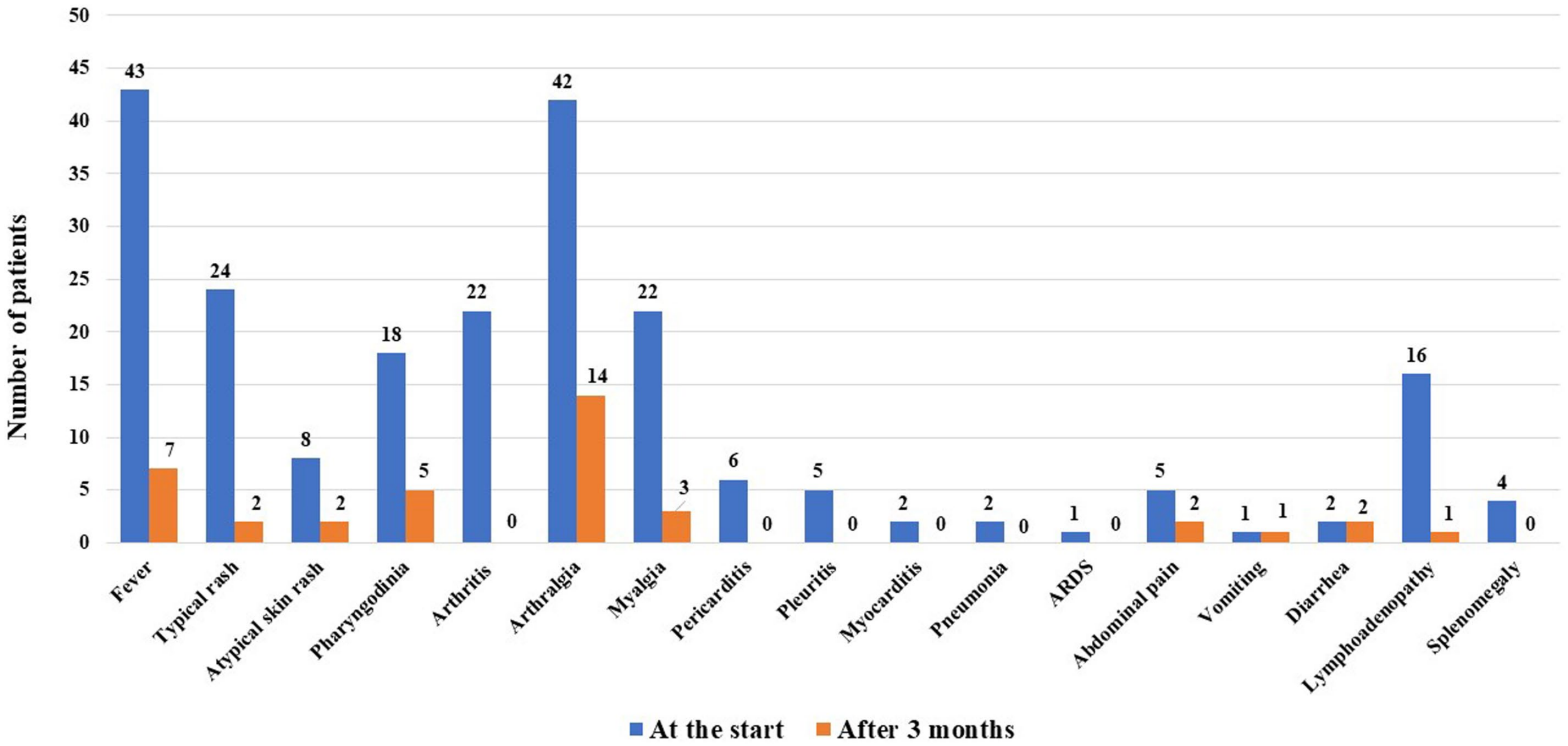
Still’s disease manifestations observed at the start of canakinumab and those persisting after 3 months of treatment.

**Figure 2:**
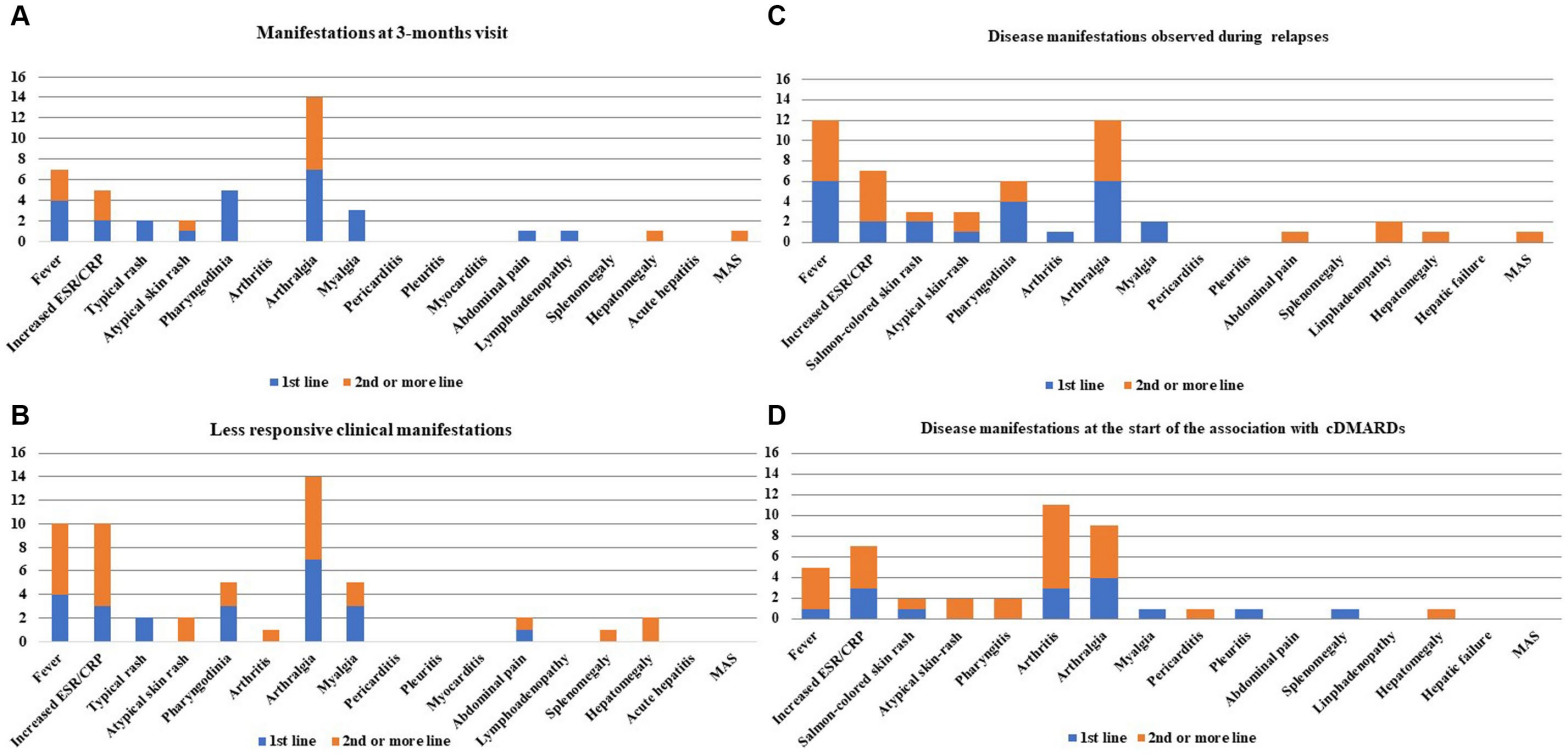
Still’s disease clinical manifestations observed at the 3-month visit **(A)**, those proving to be more resistant after the start of canakinumab (CAN) and during the entire follow-up **(B)**, those observed during relapses while on CAN treatment **(C)**, and those recorded at the time of combination with conventional disease-modifying anti-rheumatic drugs (cDMARDs) **(D)**. Clinical manifestations have been colored in order to distinguish the frequencies of patients treated with CAN as a first-line biologic agent and the frequencies drawn from patients previously treated with other biologic agents in the past. Numbers on the y-axis refer to the number of patients involved with disease manifestations.

Figure 1 shows the disease manifestations recorded at the start of CAN and those that persisted for 3 months. Figure 2A shows the clinical manifestations observed at the 3-monthly assessment, distinguishing patients according to the treatment line used.

A case of herpes zoster virus reactivation during CAN administration has been reported in the registry.

### 3.1 Treatment outcomes

**Figure 3:**
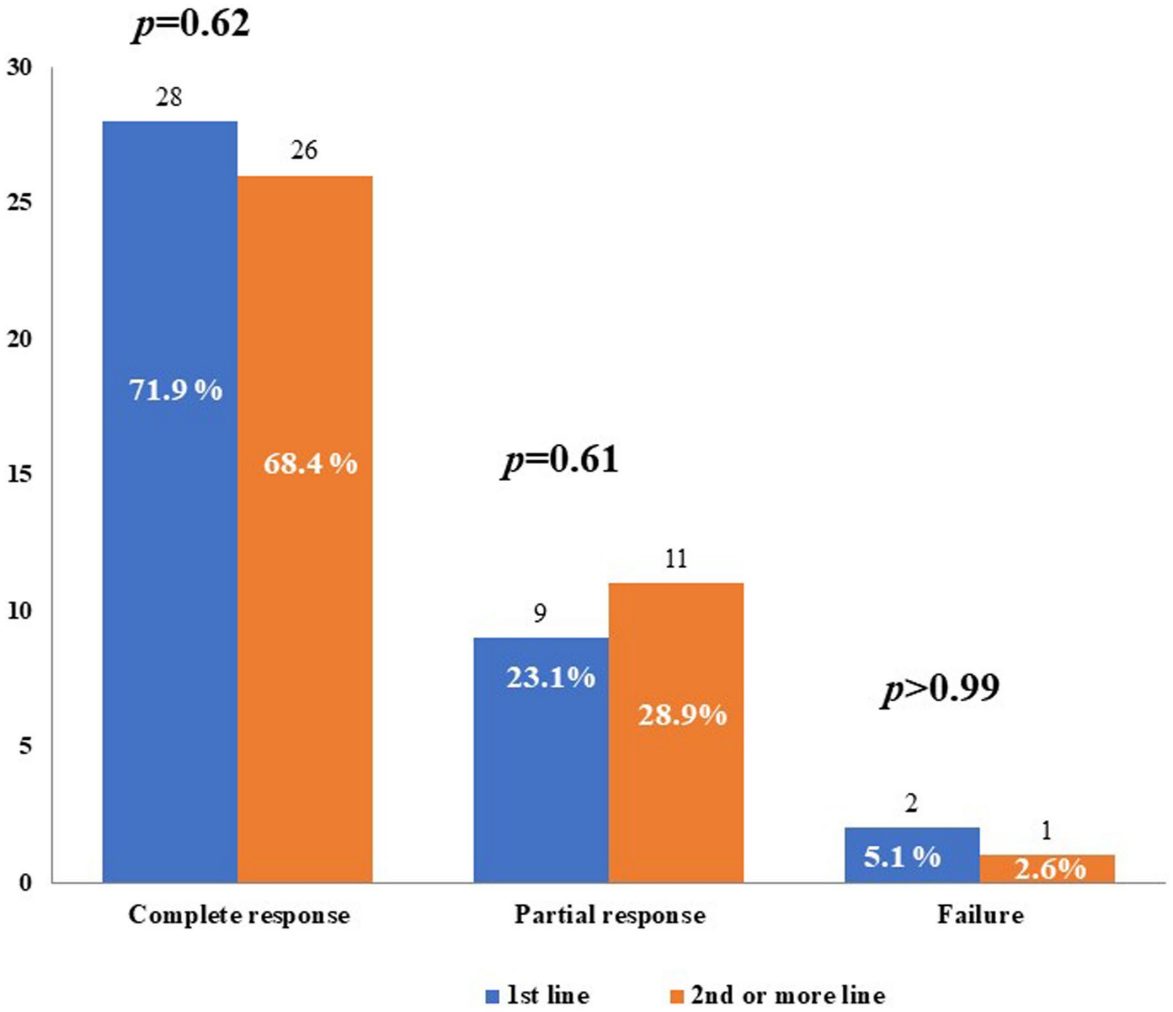
Treatment outcomes with canakinumab used as first-line biologic agent (1st line) or as second-line or subsequent biologic agent. *p*-values were obtained by using the Fisher exact test.

Figure 3 shows the described treatment outcomes of CAN when administered either as a first-line biologic agent or as a second-line or subsequent biologic agent. No statistically significant differences were observed in the frequency of complete response, partial response, or failure, according to the line of biologic treatment.

Partial response consisted of a decrease in the frequency of inflammatory relapses in one patient treated with CAN as a first-line agent, a decrease in both the frequency of relapses and severity of clinical manifestations in 13 (16.9%) patients, with 7 being treated with CAN as a first-line agent, and a decrease in the severity of both clinical and laboratory manifestations in 5 (6.5%) patients, all having been previously treated with other biologics.

**Figure 4:**
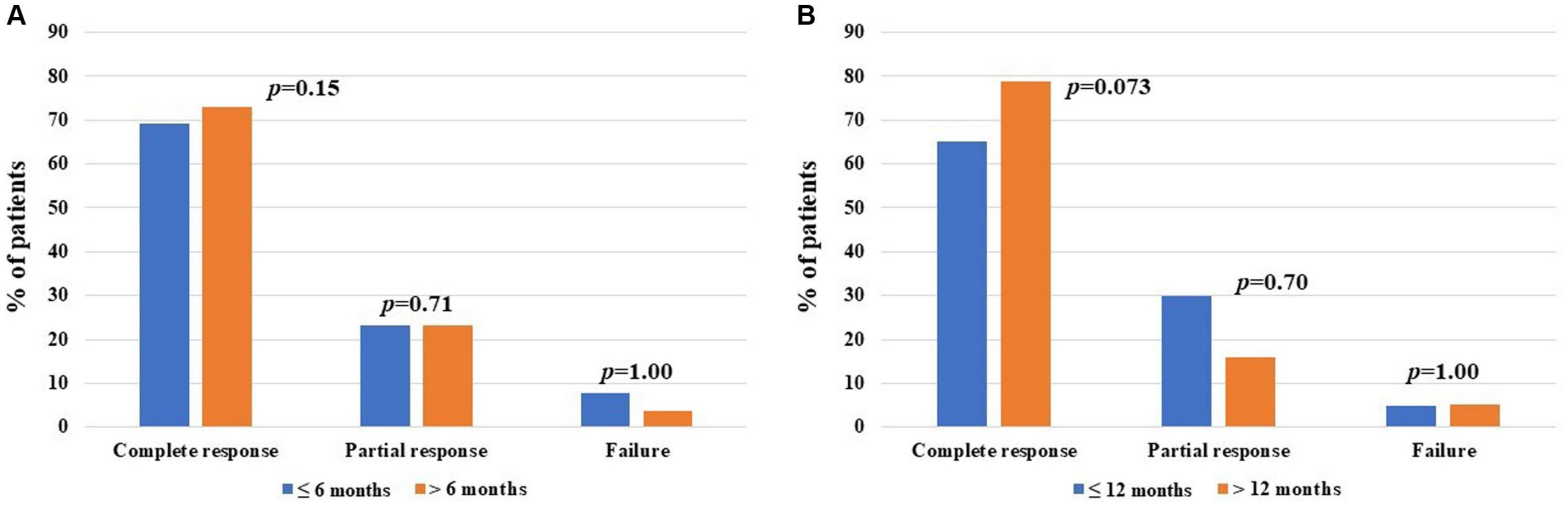
Global treatment outcomes after canakinumab (CAN) introduction among patients administered with their first-line biologic agent, distinguishing between cases starting treatment prior to and after 6 months from at the start of CAN (13 and 26 patients, respectively) **(A)** and prior to and after 12 months from at the start of CAN (20 and 19 patients, respectively) **(B)**. The *y*-axis refers to the percentage of response compared to the total number of patients included in each group; *p*-values of were obtained using the Chi-square test or the Fisher exact test, according to the frequency counts.

When comparing the frequency of complete response, partial response, and failure of CAN administration among patients undergoing their first biologic agent treatment, no statistically significant differences were observed between patients starting CAN 6 months from disease onset and those starting the treatment thereafter. Similarly, no differences were observed considering the cut-off of 12 months from disease onset. This information is reported in Figure 4.

Figure 2B shows the more recalcitrant Still’s disease-related clinical manifestations during the entire follow-up period, according to the line of biologic treatment of CAN.

During follow-up, the posology was increased in 3/39 cases being treated with their first biologic agent and in 6/38 cases previously treated with other biologic agents (*p* = 0.31). Conversely, the posology was increased in 6/39 cases treated with their first biologic agent and in 5/38 cases previously treated with other biologics (*p* = 1.00).

### 3.2 Relapses during treatment

A total of 18 (23.4%) patients experienced disease relapse during CAN treatment, with no subsequent treatment discontinuation. Nine patients were treated with CAN as a first-line biologic agent (median disease duration at the start of CAN: 6.5 months). Nine patients were treated with CAN as a second-line or subsequent biologic agent (median disease duration at the start of treatment: 18.5 months). No statistically significant differences were observed in the number of patients relapsed in the two groups (*p* > 0.99). Figure 2C shows bar charts describing the disease manifestations observed in cases of relapse during CAN treatment.

### 3.3 Treatments concomitantly associated with CAN

Non-steroidal anti-inflammatory drugs were used daily in 1 (1.3%) patient and on-demand in 6 (7.8%) patients during the CAN treatment. At the start of the CAN treatment, GCs were administered to 46 (59.7%) patients, 23 (50%) of whom were treated with CAN as a first-line biologic agent. Another 5 (6.5%) patients, all being treated with their first biologic agent, took GCs on-demand.

In the whole cohort of patients, a decrease in both the frequency of GC use (from 46 to 27 patients, *p* = 0.004) and in the mean daily dosage of prednisone (or equivalent) (38.5 ± 76.6 mg/day at the start and 7.5 ± 14 mg/day at the last assessment, *p* = 0.013) was observed. At the start of CAN, the median dosage of daily GCs was 12 mg/day (IQR: 40 mg/day) among patients treated with CAN as a first-line biologic agent and 20 mg/day (IQR: 32.5 mg/day) among patients administered their second or subsequent biologic agents (*p* = 0.63). At the three-month assessment, 30 (39%) patients continued to receive GCs (18 patients treated with CAN as a first-line biologic agent, 12 patients treated with CAN following treatment with other biologic agents, *p* = 0.24). No differences were found in the daily dose of GCs between the patients being treated with their first biologic agent (median value: 5 mg/day, IQR: 20 mg/day) and other patients (median value: 8.75 mg/day; IQR: 10 mg/day) (*p* = 0.46). At the last assessment, while on CAN, 27 (35.1%) patients were treated with daily GCs, 11 of whom were administered CAN as a first-line biologic agent (*p* = 0.34). No significant differences were found in the daily GC dosage based on the line of biologic treatment: the median GC dosage was 2.5 mg/day (IQR: 12.5 mg/day) among patients treated with CAN as a first-line biologic agent and 6.25 mg/day (IQR: 3.75 mg/day) among patients previously treated with other biologic agents (*p* = 0.47).

Four (6.5%) patients, who were all being administered their first biologic agent, were also treated with colchicine. At the start of CAN, concomitant cDMARDs were used in 18 (23.4%) patients, of whom 10 (55.6%) were treated with CAN as a first-line biologic agent. A cDMARD was added during CAN treatment in 18 (23.4%) patients, 8 on their first biologic agent, and 10 on their second or subsequent biologic agents (*p* = 0.6). Methotrexate was the most frequent cDMARD added (*n* = 15), followed by cyclosporine (*n* = 1), hydroxychloroquine (*n* = 1), and leflunomide (*n* = 1). The median methotrexate dosage administered to patients treated with CAN as a first-line biologic agent was 7 mg/week (IQR: 10 mg/week), and the median dosage was 12.5 mg/week (IQR: 8.75 mg/week) among patients treated with CAN as a second-line or subsequent biologic agent (*p* = 0.43). Figure 2D shows the manifestations requiring the start of combination therapy with cDMARDs.

Five of the 18 patients treated with cDMARDs at the start of CAN experienced a posology change, two of whom (40%) were treated with CAN as a first-line biologic agent. In four patients, a decrease in posology was recorded, and in one patient, an increase in posology was required. Five additional patients (two treated with their first biologic agent) suspended the concomitant cDMARD during follow-up due to inefficacy (*n* = 2), side effects (*n* = 1, Herpes Zoster reactivation), long-term disease remission (*n* = 1), and poor compliance (*n* = 1).

### 3.4 Reasons for CAN discontinuation

A total of 14 (18.2%) patients discontinued CAN because of lack of efficacy (*n* = 1, first-line biologic agent, treatment duration of 3 months), loss of efficacy (*n* = 4, all second-line biologic agent, median treatment duration of 12.5 months), pregnancy (*n* = 1, treatment duration of 19 months), poor compliance (*n* = 1, second-line biologic agent, treatment duration of 30 months), and long-term remission (*n* = 7, 4 with CAN as a first-line biologic agent and three with CAN as a second-line biologic agent, median treatment duration of 24 months). No difference was observed in the frequency of patients discontinuing CAN due to a lack or loss of efficacy according to the different lines of biologic treatment (*p* = 0.2).

The most frequent relapsing manifestations among patients with loss of efficacy were fever, arthralgia, and an increase in inflammatory markers (3/4 cases), followed by typical and atypical rash (2/4 cases). One patient experienced macrophage activation syndrome (MAS) and loss of efficacy.

Six of the seven patients who discontinued CAN, due to long-term remission, did not relapse thereafter (median follow-up after withdrawal: 9 months). The last patient was treated with CAN as a second agent at 36 months after discontinuation.

## 4 Discussions

CAN has been proven to be effective in controlling both clinical and laboratory manifestations of Still’s disease, regardless of the age at onset, with a complete response observed in most patients within 3 months of beginning treatment (3, 15, 17). This evidence was also confirmed in this study, as a remarkable percentage of patients experienced a complete response after CAN introduction, and only a small minority of patients withdrew from this biologic agent because of efficacy issues. Based on this large number of patients, CAN effectiveness has been proven to be even higher than reported previously (18, 19).

Notably, CAN effectiveness was not affected by the line of biologic treatment. The frequency of complete and partial responses did not change between the patients administered CAN as a first-line biologic agent and those treated with CAN as a second-line or subsequent biologic agent. Moreover, no differences were observed in the frequency of relapses during the CAN administration. Similarly, the glucocorticoid-sparing effect and management of concomitant cDMARDs were not affected by the use of other biologic agents in the past. Although all four patients who suspended CAN due to loss of efficacy were administered CAN as a second-line or subsequent biologic agent, the line of biologic treatment did not influence CAN discontinuation. Of note, many patients required the introduction of cDMARDs in both groups; in this regard, concomitant use of conventional immunosuppressants could have a role in the final treatment outcome, and this has been the focus of other studies (20). The present study confirms the results recently proposed by Alexeeva et al. (21) regarding the effective role of CAN in 46 patients with sJIA previously administered the IL-6 inhibitor tocilizumab, extending the concept to the whole spectrum of Still’s disease (both sJIA and AOSD) and confirming the results, regardless of the biologic agents previously used.

Generally, the present study indicates an overall lack of difference in the effectiveness of CAN administered in later stages compared with CAN administered earlier. This is due to the optimal effectiveness of CAN, irrespective of when it is used in terms of line of biologic treatment. Our results further encourage the use of IL-1 inhibitors in patients with Still’s disease when other biologic treatment approaches require discontinuation.

The lack of influence of the line of biological treatment of CAN seems to contradict previous studies suggesting a time window from disease onset within which the start of IL-1 inhibition could lead to better results (7, 22–26). This has mainly been suggested for anakinra administered in children, while no clear time window of opportunity has been recognized in adult patients treated with anakinra (8). A recent study conducted on 80 sJIA patients treated with CAN highlighted that the time from disease onset to receiving CAN was significantly higher among non-responsive patients 6 months after CAN introduction; however, in the logistic regression, the role of CAN treatment delay on the achievement of clinically inactive disease 6 months from the start of CAN was completely covered by the number of active joints at baseline and by a history of MAS (19).

Data from the BiKeR (Biologika in der Kinderrheumatologie) registry, a German prospective registry monitoring the biologic treatments in sJIA, an earlier start of IL-1 inhibition allowed a higher frequency of the Juvenile Disease Activity Score 10 (JADAS-10) ≤ 1 (JADAS-remission) at the last observation. The JADAS is a composite score including a physician’s global assessment of disease activity, parents’ global assessment of well-being, erythrocyte sedimentation rate (ESR), and number of joints with active disease (27). While patients’ reported outcomes (PROs) were not included in our analysis, as it is methodologically inappropriate to combine the PROs used in pediatric patients with those used in adults, no differences were observed in terms of increased inflammatory markers and frequency of arthritis or arthralgia at the 3-month assessment and at the last assessment according to the different lines of biologic treatment. However, most patients included in the BiKeR registry were treated with anakinra rather than CAN. This could lead to different results, as anakinra was reported to benefit from a window of opportunity in pediatric patients, while adult patients seem to show faster control of systemic inflammation and articular manifestations when anakinra is administered immediately after Still’s disease onset (7, 8, 22–26).

## 5 Study limitations

This study has some limitations, including the retrospective design and the relatively small number of patients involved. However, based on the rarity of the disease, the sample size achieved in this study should be considered a remarkable target and the first result of the AIDA international collaboration. In addition, adult and pediatric patients were recruited for statistical analyses. In this regard, as several studies have shown that anakinra has a window of opportunity in pediatric patients, future studies should specifically investigate whether the line of biologic treatment may specifically affect CAN effectiveness in patients with sJIA. Nevertheless, whether this effect is found in pediatric patients is insufficient to reject the null hypothesis in our cohort. Additionally, the disease activity scores and PROs used in pediatric patients are quite different from those used during adulthood. This prevented us from analyzing the variables drawn from real life. Finally, it would be interesting to differentiate patients according to the disease course (systemic versus chronic articular) and establish whether the line of biologic treatment may play a role when analyzing these two groups separately. Unfortunately, fragmenting the cohort of patients into four subgroups, according to both the line of biologic treatment and disease course would lead to the selection of samples that are too small for statistical analysis. Therefore, this should be investigated in future studies with larger cohorts of patients.

## 6 Conclusion

In conclusion, CAN demonstrated excellent efficacy in patients with Still’s disease, regardless of the line of biologic treatment, confirming its effectiveness even when administered after the discontinuation of other biologic agents.

## Data availability statement

The raw data supporting the conclusions of this article will be made available by the authors, without undue reservation.

## Ethics statement

The studies involving humans were approved by Azienda Ospedaliero Universitaria Senese. The studies were conducted in accordance with the local legislation and institutional requirements. Written informed consent for participation in this study was provided by the participants or the participants’ legal guardians/next of kin.

## Author contributions

AV: Writing – original draft. VC: Investigation, Writing – review & editing. PeS: Investigation, Writing – review & editing. LD: Investigation, Writing – review & editing. GL: Investigation, Writing – review & editing. GR: Investigation, Writing – review & editing. FT: Investigation, Writing – review & editing. IA: Investigation, Writing – review & editing. MMa: Investigation, Writing – review & editing. JS: Investigation, Writing – review & editing. AbT: Investigation, Writing – review & editing. AH-A: Investigation, Writing – review & editing. FI: Investigation, Writing – review & editing. RL: Investigation, Writing – review & editing. KL: Investigation, Writing – review & editing. HD: Investigation, Writing – review & editing. PR: Investigation, Writing – review & editing. MMo: Investigation, Writing – review & editing. HM: Investigation, Writing – review & editing. AP: Investigation, Writing – review & editing. IC: Investigation, Writing – review & editing. EM-N: Investigation, Writing – review & editing. SM: Investigation, Writing – review & editing. LS: Investigation, Writing – review & editing. RK: Investigation, Writing – review & editing. RD: Investigation, Writing – review & editing. CC: Investigation, Writing – review & editing. AlT: Investigation, Writing – review & editing. AA: Investigation, Writing – review & editing. CG: Investigation, Writing – review & editing. MT: Investigation, Writing – review & editing. EB: Investigation, Writing – review & editing. MR: Investigation, Writing – review & editing. MH: Investigation, Writing – review & editing. AL: Investigation, Writing – review & editing. IP: Investigation, Writing – review & editing. MD: Investigation, Writing – review & editing. LF: Investigation, Writing – review & editing. SB: Investigation, Writing – review & editing. LN: Investigation, Writing – review & editing. FA-O: Investigation, Writing – review & editing. GiS: Investigation, Writing – review & editing. MF: Investigation, Writing – review & editing. FCi: Investigation, Writing – review & editing. DI: Investigation, Writing – review & editing. FCr: Investigation, Writing – review & editing. PP: Investigation, Writing – review & editing. NJ: Investigation, Writing – review & editing. PK-C: Investigation, Writing – review & editing. EW-S: Investigation, Writing – review & editing. AI: Investigation, Writing – review & editing. GaS: Investigation, Writing – review & editing. PaS: Investigation, Writing – review & editing. AB: Investigation, Writing – review & editing. RG: Investigation, Writing – review & editing. GC: Investigation, Writing – review & editing. BF: Investigation, Writing – review & editing. CF: Investigation, Writing – review & editing. LC: Investigation, Supervision, Writing – review & editing.
